# BSL2-compliant lethal mouse model of SARS-CoV-2 and variants of concern to evaluate therapeutics targeting the Spike protein

**DOI:** 10.3389/fimmu.2022.919815

**Published:** 2022-07-28

**Authors:** Mohanraj Manangeeswaran, Derek D. C. Ireland, Seth G. Thacker, Ha-Na Lee, Logan Kelley-Baker, Aaron P. Lewkowicz, Paul W. Rothlauf, Marjorie Cornejo Pontelli, Louis-Marie Bloyet, Michael A. Eckhaus, Mirian I. Mendoza, Sean Whelan, Daniela Verthelyi

**Affiliations:** ^1^ Laboratory of Immunology, Center of Excellence in Infectious Disease and Inflammation, Office of Biotechnology Products, Center for Drug Evaluation and Research, Food and Drug Administration, Silver Spring, MD, United States; ^2^ Department of Molecular Microbiology, Washington University School of Medicine, St. Louis, MO, United States; ^3^ Program in Virology, Harvard Medical School, Boston, MA, United States; ^4^ Division of Veterinary Resources, Office of Research Services, National Institutes of Health, Bethesda, MD, United States

**Keywords:** SARS-CoV-2, spike protein, neurotropism, BSL-2 mouse model, pseudotyped virus, ACE2 transgenic mice, VSV (vesicular stomatitis virus), COVID-19

## Abstract

Since first reported in 2019, severe acute respiratory syndrome coronavirus 2 (SARS-CoV-2) is rapidly acquiring mutations, particularly in the spike protein, that can modulate pathogenicity, transmission and antibody evasion leading to successive waves of COVID19 infections despite an unprecedented mass vaccination necessitating continuous adaptation of therapeutics. Small animal models can facilitate understanding host-pathogen interactions, target selection for therapeutic drugs, and vaccine development, but availability and cost of studies in BSL3 facilities hinder progress. To generate a BSL2-compatible *in vivo* system that specifically recapitulates spike protein mediated disease we used replication competent, GFP tagged, recombinant Vesicular Stomatitis Virus where the VSV glycoprotein was replaced by the SARS-CoV-2 spike protein (rVSV-SARS2-S). We show that infection requires hACE2 and challenge of neonatal but not adult, K18-hACE2 transgenic mice (hACE2tg) leads to productive infection of the lungs and brains. Although disease progression was faster in SARS-CoV-2 infected mice, infection with both viruses resulted in neuronal infection and encephalitis with increased expression of Interferon-stimulated Irf7, Bst2, Ifi294, as well as CxCL10, CCL5, CLC2, and LILRB4, and both models were uniformly lethal. Further, prophylactic treatment targeting the Spike protein (Receptor Binding Domain) with antibodies resulted in similar levels of protection from lethal infection against rVSV-SARS2-S and SARS-CoV-2 viruses. Strikingly, challenge of neonatal hACE2tg mice with SARS-CoV-2 Variants of Concern (SARS-CoV-2-α, -β, ϒ, or Δ) or the corresponding rVSV-SARS2-S viruses (rVSV-SARS2-Spike-α, rVSV-SARS2-Spike-β, rVSV-SARS2-Spike-ϒ or rVSV-SARS2-Spike-Δ) resulted in increased lethality, suggesting that the Spike protein plays a key role in determining the virulence of each variant. Thus, we propose that rVSV-SARS2-S virus can be used to understand the effect of changes to SARS-CoV-2 spike protein on infection and to evaluate existing or experimental therapeutics targeting spike protein of current or future VOC of SARS-CoV-2 under BSL-2 conditions.

## Introduction

Severe Acute Respiratory Syndrome Corona Virus 2 (SARS-CoV-2) is the causative agent of Corona Virus Disease- 2019 (COVID-19) which, since its emergence late in 2019, has caused over 500 million cases of COVID-19 resulting in more than 5 million deaths and disrupted the world economy (1–3). SARS-CoV-2 infections most frequently result in mild respiratory symptoms, but 3-17% of cases develop severe pneumonia and acute respiratory distress syndrome as well as cardiovascular, renal and neurological manifestations including anosmia and ageusia, which can last for months, and 1-8% of cases result in death (3, 4). The severe morbidity and mortality of COVID-19 led to the accelerated development of vaccines and therapeutics that were first implemented under emergency use authorization and then licensed with unprecedented speed worldwide. Importantly the emergence of SARS-CoV-2 Variants Of Concern (VOC) has rendered some of these first treatments ineffective and there are lingering concerns regarding the emergence of variants resistant to the protection conferred by monoclonal antibodies (mAbs) being used as therapeutics and immunity elicited by the vaccines.

pt?>The majority of available COVID-19 vaccines and therapeutics, including monoclonal and polyclonal antibodies, target the spike protein in SARS-CoV-2 as this protein mediates binding to the human cell-surface receptor angiotensin-converting enzyme 2 (hACE2) to facilitate viral entry and infection of cells (1, 5). Neutralization potential as well as antibody-dependent cellular cytotoxicity (ADCC) are thought to be critical in predicting the efficacy of antibodies to SARS-CoV-2 (6, 7). Currently target selection is most frequently based on *in vitro* neutralization assays, which can monitor cell entry but unfortunately cannot model spread, release or transmission of the virus, or examine drug pharmacokinetics/pharmacodynamics (PK/PD), thus availability of an animal model can facilitate target selection and preclinical studies (8–10). Indeed, the increased capacity for formation of syncytia recently described for SARS-CoV-2 VOC, underscores the need for animal models in understanding correlates of efficacy and to screen and test antibodies targeting the SARS-CoV-2 spike protein (11, 12).

Several animal models have been developed to assess SARS-CoV-2 therapeutics, including in non-human primates, hamsters, ferrets, dogs and mice, but few are uniformly symptomatic, and none can be performed outside of a BSL3 facility (13–17). Availability of a model that can be used in BSL2 labs would facilitate rapid product development and testing as cost and space availability severely limit studies that require BSL3 conditions. In a previous study we reported that administration of replication competent recombinant Vesicular Stomatitis Virus (VSV) where the native glycoprotein (VSV-G) is replaced by Ebola virus glycoprotein (ZEBOV-GP) causes a lethal meningoencephalitis in neonatal mice and showed that this could be used as BSL2-compatible model to screen therapeutics for Ebola (18, 19). In this study, we use a similar strategy to develop the first symptomatic BSL-2 mouse model. We show that when mice are challenged with a pseudotyped GFP tagged vesicular stomatitis virus where the surface glycoprotein is replaced with SARS-CoV-2 spike protein (VSV-EGFP-SARS-CoV-2 (rVSV-SARS2-S)) they are infected in an hACE2 dependent maner (6). Intranasal challenge with rVSV-SARS2-S of K18-human ACE2 transgenic (hACE2tg) neonatal mice results in lung and CNS lesions. The model, which is uniformly lethal in mice, demonstrates a similar tissue tropism and lethality as SARS-CoV-2 (Washington 2020 isolate) and can be used to examine the immunoprotective effect of host immune responses and therapeutics targeting the Spike protein. Further, the model can be easily adapted to model emerging SARS-CoV-2 VOC. Lastly, the model can be safely used in BSL2 facilities as this type of construct has been used safely in vaccines (17, 19–21).

## Methods

### Animals

B6.Cg-Tg (K18-ACE2)^2Prlmn/J^ (stock 034860, hACE2tg) and C57BL/6J breeding pairs were purchased from Jackson Laboratory, bred and housed in sterile microisolator cages under 12-hour day/night cycle, in the specific pathogen-free, AAALAC accredited animal facility of the U.S. Food and Drug Administration (USFDA), Division of Veterinary Medicine (Silver Spring, MD). Pups for study were the progeny of hACE2+ male and female breeding pairs. As such, 75% of the progeny were hACE2+ as per genotyping *via* real-time PCR (primer/probe sequences available from Jackson Labs: https://www.jax.org/Protocol?stockNumber=034860&protocolID=38170). All ABSL-2 mice were handled in class II biosafety cabinets. For all ABSL-3 experiments, breeding mice were housed in ABSL-3 rated, isolated air cages with HEPA filtered output in animal rooms located in the USFDA BSL-3 facility. All studies and procedures were approved by the FDA White Oak Consolidated Animal Use and Care Committee (ACUC) and the USFDA Institutional Biosafety Committee (IBC).

### SARS-CoV-2 and rVSV-SARS2-S virus preparation

SARS-CoV-2/human/USA/USA-WA1/2020 and an infectious clone of this virus expressing EGFP (for fluorescence imaging) were obtained from ATCC through Tony Wang (CBER, FDA). Variants of concern, including SARS-CoV-2: B.1.1.7 (NR-54000), B.1.1.351 (NR-54008), P.1 (NR-54982) and B.1.617.2 (NR-55611) were acquired from BEI Resources. VSV-eGFP-SARS-CoV-2 recombinant viruses (rVSV-SARS2-S), expressing Spike proteins of the Wuhan isolate (GenBank MN908947.3), B.1.1.7 (α), B.1.351 (β) and P.1 (γ) and B.1.617.2 (Δ) were prepared by Dr. Sean Whelan (6). VSV expressing the Wuhan isolate Spike encodes the full length Spike gene, which acquired a mutation that truncated the cytoplasmic tail by 21 amino acids, as described; the Spike genes of B.1.1.7, B.1.351, P.1 and B.1.617.2 viruses were cloned to truncate the final 21 amino acids of the Spike gene prior to rescue. All viruses were propagated (fewer than 3 passages) in Vero E6 cells to generate master stocks. The stocks were titrated (TCID_50_) on Vero E6 cells and working stocks were aliquoted and stored at -80°C.

### Virus infection and organ collection

Neonatal hACE2tg pups Post natal day 1 (P1) or Post natal day 5 (P5) as indicated in each figure, were infected with 1.0 x 10^5^ TCID_50_ or the indicated challenge dose of virus by intranasal inoculation. A 10 μl pipet tip was used to administer the virus preparation to the nostrils and pups inhaled the droplets (5-10 μl total volume). Due to the young age of the pups, minimal restraint and no anesthesia was required for inoculation. The female breeder was left in the cage throughout the experiment to provide nourishment and care to the pups. At the indicated times post-infection, mice were euthanized by CO_2_ asphyxiation and exsanguination by transcardiac perfusion with PBS prior to collecting the organs. Failure to gain weight compared to C57Bl/6 mice, difficulty breathing, and activity level in the cage were scored from normal (0,-/+) to severe (3, +++). Clinical progression was assessed by observing the mice in the cage as well as individually. Changes in behavior were recorded and tallied. Per protocol, any animal that was unresponsive to touch stimuli were considered moribund and euthanized. In addition, animals that lost more than 20% of their body weight were euthanized. Lastly, animals with symptoms that prevent accessing nutrition, were euthanized as well. Tissues for gene expression analysis were collected and flash frozen in liquid nitrogen and stored at -80°C until processing. Tissues for immunohistochemistry were placed in 4% paraformaldehyde (PFA) for fixation for 24 hours. For histology samples, animals were perfused with 4% PFA. A 25 G needle was inserted in the trachea to expand the lungs with 4% PFA prior to collection. The samples were stored in fixative and sent to Histoserv labs (Germantown, MD) for paraffin embedding, sectioning and hematoxylin and eosin (H&E) staining.

### Virus quantification

Absolute quantification was determined using full length SARS-CoV-2 spike RNA transcribed from pcDNA 3.1- SARS2-Spike plasmid using the T7 Megascript transcription kit (ThermoFisher, Carlsbad, CA). pcDNA3.1-SARS-2-Spike was a gift from Fang Li (Addgene plasmid #145032). The number of copies transcribed was calculated based on absorbance at 260 nm. One-step reverse transcription (RT)-real time qPCR was performed using the RNA-to-Ct kit (Thermofisher, Carlsbad, CA). A 10-fold serial dilution of transcribed RNA was used to generate a standard curve for quantification. Total RNA was isolated from tissues homogenized in Trizol (Invitrogen) per manufacturer’s instructions. One microgram (µg) of isolated total RNA from infected tissues was analyzed in each reaction. This assay amplifies the region corresponding to nucleotide 2442-2500 of SARS-CoV-2 spike gene. Forward primer: GAGGTCATTTATTGAAGATCTACT; Reverse Primer: GCAATCACCATATTGTTTGAT; Probe: AAAGTGACACTTGCAGATGCTGGCTT. Primers were synthesized through Genescript (Piscataway, NJ). Cycling conditions: 48 °C for 15 min, 95 °C for 10 min, 40 cycles of 95 °C, 15 sec and 60 °C for 1 min. Data was collected and analyzed using an Applied Biosystems Viia7 real-time instrument with QuantStudio Software (ver. 1.6).

Tissue culture Infectious Dose (TCID) 50 assay: Infectious SARS-CoV-2 and rVSV-SARS2-S levels in organ homogenates (half brain or two lung lobes in 1 mL of culture medium) from perfused mice were measured on Vero E6 monolayers using an end-point dilution assay as previously described; TCID_50_ values were calculated using infectivity calculator based on Reed and Muench method (22, 23).

### Neutralization assay

Vero E6 cells were seeded at 2-4 x 10^4^ cells/well in 96 well plates and incubated at 37°C to reach 70-80% confluency. Antibody samples were incubated with 1.0 x 10^3^ TCID_50_ of virus for 1 h at 37°C. Antibody-virus complexes were added to Vero E6 cell monolayers in 96-well plates and incubated at 37°C for 1 h. Subsequently, the cells were washed, and incubated for 12-16 h in fresh media. Neutralizing ability of antibodies was assessed by monitoring levels of GFP positive cells in the infected wells and compared to untreated control wells using an Olympus IX-81 microscope. GFP levels were monitored using Victor X3 multimode plate reader. Anti-Spike protein (RBD) mAb CV30 (mouse IgG1, kappa) was obtained from Absolute antibody (Boston, MA, product code: Ab02019-1.1). Heat inactivated plasma samples were obtained from Washington Adventist Medical Health care and Shady Grove Adventist hospital. Plasma from 3 donors who recovered from SARS-CoV-2 infection in April-May 2020 before the emergence of VOCs and had high neutralizing antibody titers were pooled and used as Human Immune plasma for the neutralization assay and protection studies in mice.

### Gene expression analysis using Nanostring

Mouse Immunology Panel (Nanostring Technologies, Seattle, WA) consisting of 561 genes including 15 internal reference genes was used. The complete gene list for the nCounter Mouse Immunology panel used in this experiment is available at www.nanostring.com. Reporter and Capture probes were mixed with 100 ng of total RNA (20 ng/μl in molecular grade, ultra-pure ddH_2_O) isolated from the CNS and lungs of uninfected, SARS-CoV-2 infected and rVSV-SARS2-S infected hACE2tg mice. RNA and probes were hybridized for 18 hours prior to processing and data acquisition using the Nanostring MAX acquisition system, using ultra-sensitive mode and counting 280 fields of view (FOV) per sample. These data were normalized to internal controls and housekeeping genes in the panel as per manufacturer’s instructions. For **Figure 4A**(left panel) as well as for **Supplementary Figures 4** and **7**, genes and samples were sorted using hierarchical clustering and plotted using the iheatmapr library. Note that in these figures, log-scale counts for each of the samples were normalized within each gene by calculating their Standard Score, which was defined as the difference between the logscale counts sample and the average logscale count divided by the standard deviation of the logscale counts for that same gene. Thus, the colors ranging from white to green indicate the relative expression of the gene, rather than suggest up or down regulation. In radial graphs (**Figure 4B**), gene expression is expressed as fold-change in gene expression, relative to age-matched uninfected control animals. These data were analyzed using Nanostring nSolver (ver. 4) and plotted using GraphPad Prism (ver 9.x). Dendrograms were created in R (v 4.1) (24) using the iHeatmapr (25) library.

Pathway analysis: Gene expression profile in the brains of mice infected with SARS-CoV-2 (red) and rVSV-SARS2-S (blue) was explored using GAGE analysis (26) on GO (Gene ontology) pathways. Stat.mean (bars) is the average of individual statistics for the gene set of each pathway, with bars representing direction and magnitude of the pathway. The Q-values (lines) show the False Discovery Rate (FDR) adjustment of the global p-value derived from individual tests as performed by GAGE analysis. Shown is a curated list of the most up and down-regulated pathways, plotted using the ggplot from the tidyverse packages (27). For the dendrograms, the signal was standardized for all sample’s log-scaled counts across a given gene.

### Immunofluorescence immunohistochemistry and histology

One hemisphere of the brain and both lungs from infected and uninfected animals were fixed with 4% PFA and sent to Histoserv (Germantown, MD), where the tissues were embedded in paraffin and sectioned. Sections were stained with hematoxylin and eosin (H&E) by Histoserv, and unstained sections returned to the lab for immunohistochemical staining. These sections were deparaffinized using xylenes, and rehydrated in graded ethanol (100%, 95%, 70%, 50%). Sections were rinsed in ddH_2_O and underwent antigen retrieval in 0.1M sodium citrate (pH 9.0) for 8 minutes, then allowed to cool at RT for an additional 10 minutes and washed with PBS. Endogenous peroxidase activity was removed by treating the sections with 3.7% H_2_O_2_ for 30 minutes. After washing with PBS the tissue sections were permeabilized using 0.5% Triton X-100 in PBS for 60 minutes at RT and blocked with 5% normal goat serum (NGS) and 1% bovine serum albumin (BSA) in PBS + 0.05% Triton X-100 for 1 hour. Staining was performed using a three-step detection, with: anti-SARS-CoV-2 Spike RBD rabbit polyclonal antibody (Sino Biologicals, Wayne, PA, Cat #40150-T30) for 2 hours at RT, followed by a biotinylated anti-rabbit IgG for 1 hours at RT and Streptavidin-HRP for 30 minutes (Vector Laboratories, Burlingame, CA). Immunostaining was detected using ImmPACT DAB peroxidase substrate and washed as per manufacturer’s instructions ((Vector Laboratories, Burlingame, CA). Stained sections were dehydrated in graded ethanol (70%, 95%, 100%), cleared with histology grade xylenes, and mounted with DPX mountant (MilliporeSigma, St. Louis, MO).

To characterize the CNS infection by IF-IHC, one hemisphere of brains from infected and uninfected animals were placed in freshly prepared 4% paraformaldehyde (PFA) solution (in 1 x PBS pH 7.4) immediately after extraction (RNA was extracted from the other hemisphere as described above). After 24 hours of fixation at 4°C, the tissue was transferred to 30% sucrose in 1 x PBS for cryoprotection. The brains remained in 30% sucrose at 4°C until they sank in the solution (typically ≥ 24 hours). Sucrose embedded tissue was mounted in TissueTek O.C.T (Sakura-Finetek, Torrance, CA) and frozen. The brains were stored at -80°C until sectioned. Sagittal sections (25 μm thick) were cut using a Leica CM1900 cryostat at a chamber temperature of -20°C (Leica Biosystems, Buffalo Grove, IL) and mounted onto Superfrost-Plus Gold microscope slides (Fisher Scientific, Carlsbad, CA). Mounted sections were stored at -20°C until staining. Prior to staining, sections were warmed to room temperature (RT) to dry, then immersed in phosphate buffered saline (PBS). Antigen retrieval was then performed as described above. The sections were then washed with PBS and permeabilized using 0.5% Triton X-100 in PBS for 60 minutes at RT; then blocked and autofluorescence quenched with 5% normal goat serum and 1% bovine serum albumin (BSA) in PBS + 0.05 % Triton X-100 and 0.3M Glycine for 2 hours. Primary antibodies used include: anti-SARS-CoV-2 Spike RBD rabbit polyclonal antibody (Sino Biologicals, Wayne, PA, Cat #40150-T30), neurofilament heavy chain cocktail (SMI31, Cat # 801601) and SMI32 mAb (Cat #40150-T30), Biolegend, rat anti-CD45 (BD Biosciences, Clone 30-F11, cat# 553076), rabbit anti-Ionized calcium-Binding Adapter molecule-1 (Iba-1) (Wako, Cat#019-19741). Endogenous EGFP expressed by the viruses was detected without additional staining. Tissue sections were incubated overnight in a humidified chamber at room temperature with primary antibodies diluted with 1% BSA in PBS + 0.5% Triton X-100. The slides were then washed with PBS + 0.05 % Triton X-100 followed by 1 x PBS and treated with the appropriate AlexaFluor (AF)-conjugated secondary antibodies (raised in goat) (ThermoFisher, Carlsbad, CA), diluted in 1% BSA in PBS + 0.05% Triton X-100 for > 120 min at RT. All IF-IHC sections were mounted with Pro-Long Diamond anti-fade mounting media containing DAPI (ThermoFisher, Carlsbad, CA). Sections were imaged using an Olympus VS-120 virtual slide microscope (Olympus LSS) using a 20x or 40x objective lens. AF-labelled antibodies and endogenous fluorescence were detected at emission wavelengths: 405 nm (DAPI), 535 nm (Alexa-fluor 488, EGFP), 605 nm (Alexa-fluor 568) and 650 nm (Alexa-fluor 647). Regions of interest were captured using VS-Desktop software (ver 2.9, Olympus LSS). For confocal imaging, images were acquired using a Zeiss LSM 880 confocal microscope, using 405 nm (DAPI), 488 nm (AF488, EGFP), 561 nm (AF568) and 633 nm (AF647) excitation lasers. Optimal fluorescence detection settings were determined and applied to all sections equally. Images were acquired using the Zeiss Zen software. Maximum intensity projections of acquired Z-stacks were compiled using Zeiss Zen and/or ImageJ + Fiji (NIH) software (28). Images were compiled into figures using Adobe Photoshop CS software.

### Statistics

Weight gain was analyzed using a Kruskal Wallis test. Survival experiments were analyzed using the log-rank test. Two-tailed unpaired Student’s t test or 1-way ANOVA with Dunnett’s multiple comparisons were used as appropriate. Statistical analyses were conducted with GraphPad Software (version 7.03). P < 0.05 was considered statistically significant. Graphs show Mean and SD.

## Results

### The model: SARS-CoV-2 spike pseudotyped VSV infects human ACE2 transgenic mice and causes disease comparable to SARS-CoV-2 infection

Previous studies established that K18-hACE2 transgenic mice (hACE2tg), in which hACE2 expression is driven by the epithelial cell-specific human cytokeratin 18 (K18) promoter and expressed in airway epithelial cells, as well as brain, liver, kidney, spleen, heart, and intestine renders mice susceptible to SARS-CoV-2 infection, however these challenge studies have to be performed in BSL3 labs (13, 29–32). To develop a BSL-2 mouse model suitable to test therapeutics targeting SARS-CoV-2 spike protein, we challenged hACE2tg mice (4-6 weeks old) with a replication-competent recombinant enhanced green fluorescent protein tagged VSV where the native glycoprotein was replaced by SARS-CoV-2 spike protein (rVSV-SARS2-S). Neither intramuscular (IM) nor intranasal (IN) infection of adult hACE2tg mice with rVSV-SARS2-S (up to 1 x 10^5^ TCID_50_/mouse) resulted in weight loss or symptoms (**Supplementary Figure 1**) and no viral RNA was detectable in blood at 3 dpi, or blood, liver, lung or brain homogenates at 8 dpi. This suggests that rVSV-SARS2-S could not be used to model COVID-19 in adult hACE2tg mice.

**Figure 1:**
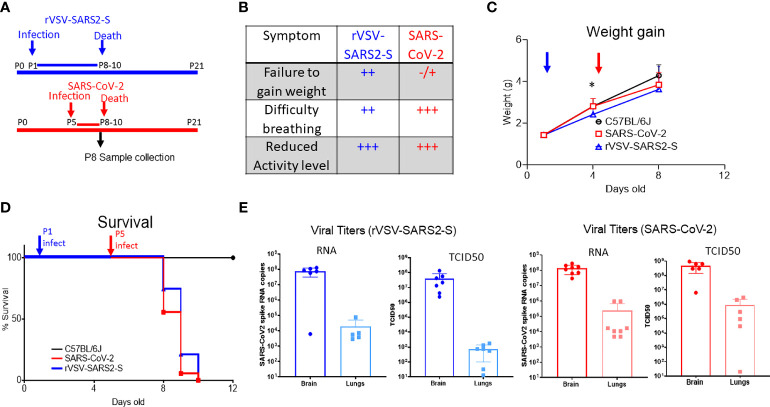
Infection of hACE2tg mice with rVSV-SARS2-S or SARS-CoV-2 results in infection of lungs and brain and leads to lethal disease. **(A)** Schematic representation of the timeline of SARS-CoV-2 and rVSV-SARS2-S infection and disease course. Note that mice were challenged on P1 with rVSV-SARS2-s or on P5 with SARS-CoV-2 but tissues were collected on P8 for both groups. **(B)** Symptoms in hACE2tg mice infected with SARS-CoV-2 and rVSV-SARS2-S viruses. **(C, D)** Control mice (C57BL/6J, black, circle n > 10, overlapping for both viruses) or hACE2tg mice (blue and red) were challenged intranasally with 10^5^ TCID_50_ of SARS-CoV-2 (red squares, n > 10) or rVSV-SARS2-S (Blue triangles, n > 10) and monitored for weight gain **(C)** or survival **(D)**. **(E)** Viral RNA titers in lung and brain homogenates of mice infected with rVSV-SARS2-S infected (n = 5/group) and SARS-CoV-2 infected (n = 5-8/group) as assessed by SARS-CoV-2 spike protein specific Taqman assay and infectious virus measured by TCID_50_ assay in Vero E6 cells. Figure shows titers per ug of RNA (qRT-PCR) or half-organ (TCID_50_). Graphs show mean ± SD; *denotes weight difference between rVSV-SARS2-S and uninfected mice at P8 (p<0.05) as measured by Krusckal-Wallies non parametric ANOVA.

Neonatal mice have increased susceptibility to viruses and develop symptomatic infections when challenged with Alpha-, Arena- and Flavi-viruses, even when the adults of the same strain are resistant (18, 19, 23, 33–35). As expected, neonatal hACE2 tg mice succumbed following a SARS-CoV-2 (10^2^-10^5^ TCID_50_ Washington 2020 isolate, IN) challenge in a dose-dependent manner (**Figures 1A–D** and **Supplementary Figure 2F**) and high titer virus were evident in the lungs and brain of mice challenged IN with 10^5^ TCID_50_ as determined by viral RNA levels and TCID_50_ (**Figure 1E**). To determine whether VSV-SARS2-S would cause a symptomatic infection in neonatal hACE2tg mice, P1 mice were challenged with rVSV-SARS2-S (10^2^-10^5^ TCID_50_ IN; **Figures 1A–D** and **Supplementary Figure 2C**). While the disease progression was slower than with SARS-CoV-2, all mice showed reduced weight gain starting by 3 dpi, developed dyspnea and reduced exploratory activity by 6DPI, and succumbed by 9 dpi (**Figures 1B–D**). Assessment of viral distribution in the hACE2tg mice showed that both models had higher levels of viral RNA and infectious virus in the brain, and lower but consistent levels in the lungs (**Figure 1E**). Viral RNA levels in blood, liver, spleen, heart, kidney, and small intestine of mice challenged with rVSV-SARS2-S were negligible and infectious virus was not detectable by TCID_50_ assay (**Supplementary Figure 3A**). Indeed, these were not different from those reported in adult hACE2tg mice (29, 36), although the relative viral load can change significantly depending on the day it is assessed (32). Although infectious virus was only detected in the brain and lungs of neonatal hACE2Tg mice, expression of human ACE2 was seen in all the tissues tested (**Supplementary Figure 3B**) and the expression pattern was similar to that found in adult hACE2Tg mice (30, 32). Neonatal C57BL/6J mice challenged with SARS-CoV-2 or rVSV-SARS2-S showed no change in weight or lethality and no virus was recovered at P4 or P9. Surprisingly, even neonatal Interferon alpha and beta receptor 1 Knock-out mice (C57BL/6J-IFNAR KO) showed no weight change or symptoms indicating that rVSV-SARS2-S, like SARS-CoV-2, requires the presence of hACE2 to infect cells (**Supplementary Figure 2**). Concordantly, *in-vitro* studies with BHK-21 cells demonstrated that rVSV-SARS2-S requires the presence of hACE2 to infect cells (**Supplementary Figure 3C**). In summary, the comparable tissue tropism, disease phenotype and lethality of SARS-CoV-2 under BSL-3 conditions and the rVSV-SARS2-S infection suggested that this is a viable surrogate model for SARS-CoV-2 spike mediated infection under BSL-2 conditions to test therapeutics and vaccines directed to the spike protein.

### Lung infection

We next compared the infection and response in the lungs. Given the difference in disease progression, studies were performed by challenging with SARS-CoV-2 at P5 and with rVSV-SARS2-S at P1 and then collecting tissues for both models on P8, when most animals were symptomatic but not moribund (**Figure 1A**).

**Figure 2:**
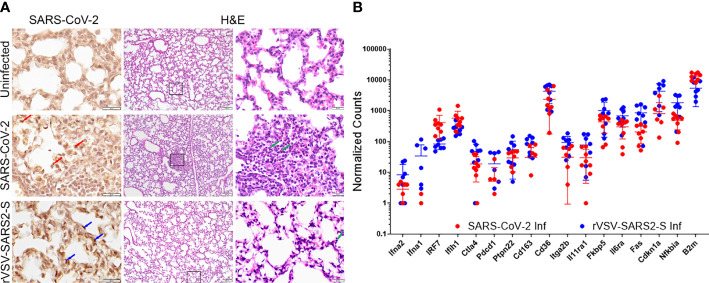
Lung infection in mice challenged with SARS-CoV-2 and rVSV-SARS2-S viruses. **(A)** Left panels: Immunohistochemistry for Spike RBD. Red arrows indicate SARS-CoV-2 infected cells. Blue arrows indicate rVSV-SAR2-S infected cells. Right panels show H&E staining of lungs (100 and 600X). Green arrows indicate infiltrating immune cells (image representative of 6 mice/group). **(B)** Fold-change in selected genes from the lungs of SARS-CoV-2 and rVSV-SARS2-S (n=8) infected mice at P8 compared to age-matched uninfected control mice as assessed using Nanostring technology (nCounter Mouse Immunology Panel). The complete gene expression profile can be found in **Supplementary Figure 4**.

In both models, on P8, infected mice showed minor discrete lesions characterized by small dispersed inflammatory foci and alveolitis with minor polymorphonuclear (PMN) leukocyte infiltration PMN infiltration and blood extravasation into the alveolar space (**Figure 2A**). Consistent with this there was a moderate increase in the mRNA expression of genes coding for interferon responses (Ifna1, Ifna2, Ifih1, Irf7, and Ptpn22) as well as platelet markers CD36 and Itga2b. However, while the changes in mRNA expression for proinflammatory signals were modest, the increase in Il6ra and Il11ra1, was not paired with an increase in Il6st and there was no significant increase in Il6 or Tnf indicating that the inflammation in the lungs is minor (**Figure 2B**, **Supplementary Figure 4**). Of note, the increased level of Fkbp5 known to regulate NFkB and RIG-I signaling could contribute to the muted response (37). Concordant with the minor inflammatory response in lungs, there was no significant signal of inflammation in sera (**Supplementary Figure 5**).

### CNS infection

**Figure 3:**
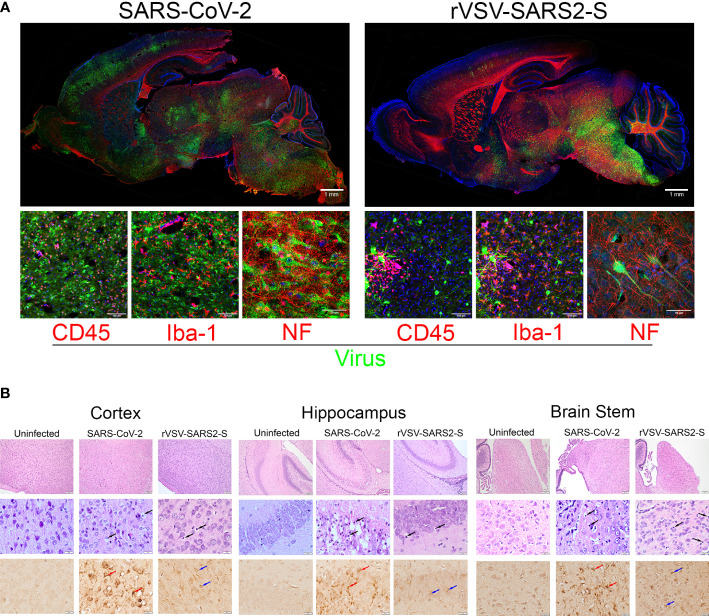
Neuroinvasion and tissue damage in SARS-CoV-2 and rVSV-SARS2-S infected mice. **(A)** Top: Whole brain, sagittal section of P8 mice infected with SARS-CoV-2 and rVSV-SARS2-S viruses. Replicating virus labeled with GFP (green), Neurofilament (Red), and nuclei with DAPI (blue). Scale bar:1mm. Bottom: Confocal images of cerebral cortex stained with green (GFP expressed by infecting virus) and Red Indicating: CD45 (infiltrating immune cells), Iba-1 (microglia) or NF (neurons) (N = 6). Scale bar: 25μm. **(B)** H&E and Spike RBD IHC from infected regions of the CNS. Top: wide-field H&E. Middle: high magnification H&E. Black arrows indicate degenerating neurons. Bottom: IHC staining for Spike RDB. Red arrows indicate SARS-CoV-2 infected cells. Blue arrows indicate rVSV-SAR2-S infected neurons in these regions (N = 6). Scale bar: 100 and 20 μm.

**Figure 4:**
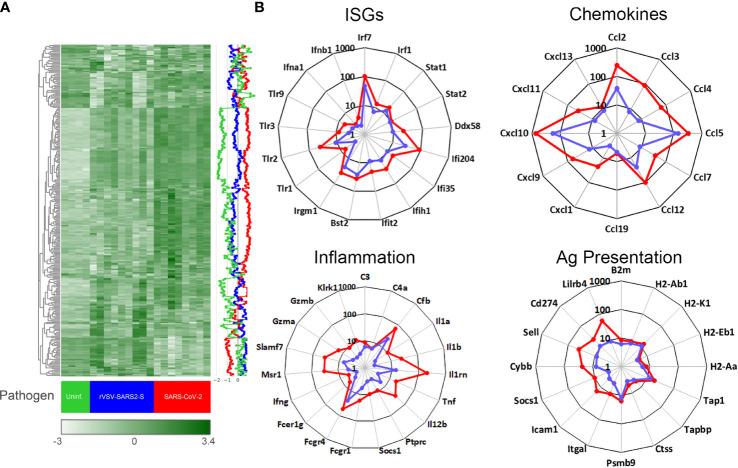
Gene expression in the brain of mice infected with SARS-CoV-2 and rVSV-SARS2-S. **(A)** Nanostring mouse immunology panel. Heatmap shows gene expression (normalized counts as described in materials and methods) in the brain of SARS-CoV-2 infected (red), rVSV-SARS2-S infected (blue) and uninfectected control (green). Each column in the heatmap represents an individual mouse (n = 4-8/group; P8) and the blue, red and green lines on the right depict the average gene expression. The complete labeled Nanostring panel of 547 immunology-related mouse genes used for analysis is presented in **Supplementary Figure 7**. **(B)** Changes in gene expression (selected) in brain tissue of SARS-CoV-2 (red) or rVSV-SARS2-S (blue) infected mice relative to age-matched uninfected mice. Data is presented as fold change in infected, compared to average of uninfected age-matched controls. Note that gene expression in **(A)** is reported as normalized absolute counts and gene expression in **(B)** is reported as fold increase over age-matched uninfected control mice.

In both models, the viral load was highest in the brains. Visualization of the virus at P8 using GFP-tagged virus showed significant spread of the virus throughout the brainstem, subcortical nuclei, mid and hind brain in both models (**Figure 3A**). In the cortex, there was evidence of virus in neurons and neurites spanning multiple layers (**Figure 3A**). Colocalization with neurofilament (NF+) suggests that neurons were infected while the surrounding microglia (Iba+) were uninfected but display activated morphology (**Figure 3A** and **Supplementary Figures 6A, B**). Further, in both models there was an increase in degenerative neurons, including in the olfactory bulbs (**Supplementary Figure 6A**) suggesting a possible path for the virus to enter the CNS. Of note, at P8 the virus in the brains of SARS-CoV-2 infected mice was more broadly distributed compared to those of rVSV-SARS-CoV-2-S infected mice (**Figure 3A**). Differences were also notable in cerebellum where SARS-CoV-2 infects Purkinje cells, while rVSV-SARS2-S infected neurons in the molecular and granular layers as well as in the white matter tracts (**Supplementary Figure 6A**). Associated with the broader virus distribution, there is a greater infiltration by CD45+ immune cells in the CNS of SARS-CoV-2 infected mice, which was confirmed by H&E (**Figure 3B**) and by the higher expression of Ptprc (CD45) in the infected CNS (**Figure 4**). These differences were consistent with the accelerated disease progression in the SARS-CoV-2 model.

Unlike the lungs, the gene expression screen for the CNS from both models harvested on P8 (3 dpi for SARS-CoV-2 and 7 dpi for rVSV-SARS2-S) showed a clear change in mRNA expression patterns with an upregulation of inflammatory genes consistent with viral encephalitis (**Figure 4**). Pathway analysis showed both models inducing strong upregulation of genes linked to innate immune, type I interferon, antiviral and chemokine activity, as well as downregulation of genes in the tissue remodeling, cell development, erythropoiesis, and neurogenesis (**Supplementary Figure 8**). More specifically, there was strong upregulation of Irf-7, Irf-1, Stat-1, Stat-2, Ddx58 and Ifih1 as well as Ifi35, Ifit2, Ifi204, Bst-2 and Irgm-1 indicative of a strong type I interferon response to the virus while increased transcript levels of complement factors C3, C4a and Cfb suggests complement pathway activation (**Figure 4**). Elevated levels of Ptprc (Cd45) are consistent with an increase in infiltrating cells and the increase in Cxcl10, Ccl5, Ccl12 and Ccl2 as well as B2m, MHC class I and class II, Tap1, Tapbp, Ctss, and Psmb9 indicate increased antigen processing and presentation. However, a weak upregulation of Il1, Il12 and Tnf, and increased expression of Il1rn, Socs1, CD274 (Pdl1) and Lilrb3/4 suggests local immune regulation (**Figure 4** and **Supplementary Figure 7**. Thus, while the overall pattern of mRNA expression in the CNS elicited by SARS-CoV-2 and rVSV-SARS2-S were comparable, the magnitude of the response was higher in SARS-CoV-2 infected mice.

### Testing therapeutic antibodies

**Figure 5:**
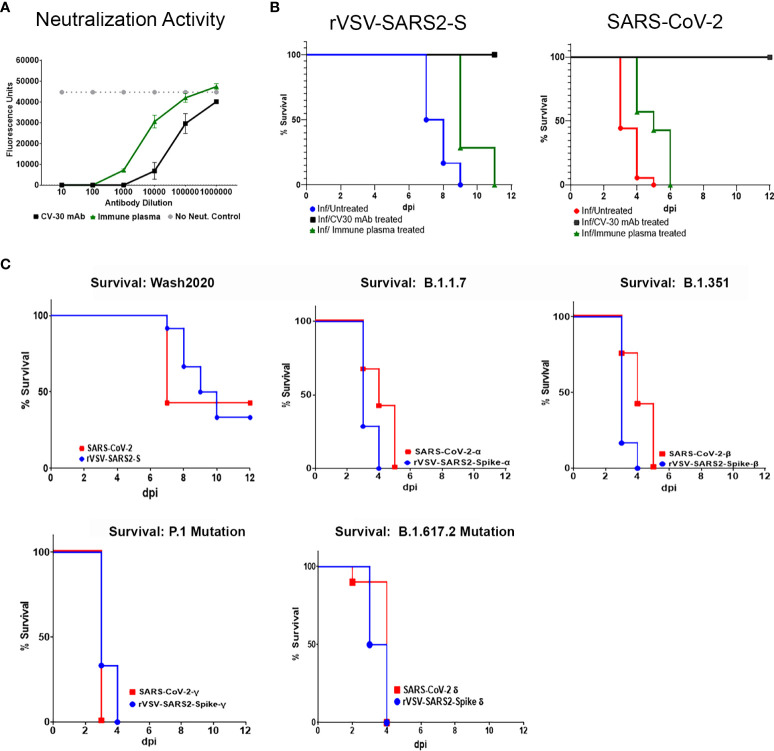
*In vitro* neutralization and *in vivo* modeling of antibodies to SARS-CoV-2 Spike bioactivity. **(A)** Neutralizing activity of an anti-SARS-CoV-2 RBD domain mAb (Black square) and human immune serum pooled from donors recovered from SARS-CoV-2 infection (green triangles) were tested in a micro neutralization assay on Vero E6 cells infected with replicating rVSV-SARS2-S. **(B)** Human ACE2tg mice were treated intraperitoneally with 2 µg/g of CV30 mAb (black line) or 5 µl/g of human immune serum (green line) 24 hours before being intranasally infected with 10^5^ TCID_50_ of rVSV-SARS2-S (n ≥ 7) or SARS-CoV-2 and monitored for survival (n ≥ 7/group). Untreated, age-matched SARS-CoV-2 (red line) and rVSV-SARS2-S (blue line) Infected hACE2tg mice were used as controls. Differences in survival were assessed using a Log Rank Mantel Cox test. ** p < 0.005. **(C)** Human ACE2tg mice were infected at P5 with 10^4^ TCID_50_ SARS-CoV-2 or 10^5^ rVSV-SARS2-S (10^4^ TCID_50_ of rVSV-SARS2-S shown in **Supplementary Figure 2C**), or with 10^4^ TCID_50_ of SARS-CoV-2 Variants of Concern (SARS-CoV-2^α,^ SARS-CoV-2^β^, SARS-CoV-2^γ^SARS-CoV-2^Δ^) or VSV pseudotype virus expressing the corresponding SARS-CoV-2 spike protein and monitored for survival (n = 6-13 mice/group).

Antibodies targeting the spike protein are being used to treat infected patients with high clinical risk. The neutralizing capacity of these antibodies is often tested *in vitro* by pre-mixing the antibodies with a non-replicating pseudotype virus and then measuring cell entry into susceptible cells such as Vero E6. The use of replicating viruses can improve the assessment as it reflects not only entry, but replication and release of the virus from the cells. Here, we tested human immune plasma (from patients who recovered from SARS-CoV-2 infection) and anti-SARS-CoV-2 spike protein RBD domain monoclonal antibody (CV-30). Both antibodies neutralized rVSV-SARS-CoV-2-S virus (**Figure 5A**). Availability of animal models infected with replicating virus can provide evidence of *in vivo* efficacy of these therapeutics. As shown in **Figure 5B** treatment with CV-30 (2μg/g) administered intraperitonealy conferred 100% protection from lethal challenge while human immune plasma (5μL/g) failed to protect mice from lethal infection (**Figure 5B**). The difference in bioactivity *in vivo* correlated with the relative differences in neutralizing titer of the 2 preparations. However, the same dose of CV-30 mAb treatment post-infection did not rescue hACE2tg mice from lethal infection in either model (**Supplementary Figure 9**). Importantly, the effect of the different antibodies tested prophylactically or therapeutically mirrored each other in SARS-CoV-2 and rVSV-SARS2-S models, demonstrating that the BSL-2 model can be a suitable option to test antibodies designed to prevent SARS-CoV-2 spike protein interaction with ACE2 expressing cells and a useful tag to monitor virus spread in tissues.

SARS-CoV-2, like other RNA viruses, is prone to genetic evolution, and even single amino acid changes can drastically affect a virus’s ability to bind ACE2 or evade the immune system (5, 38). Over the last year, several variants of concern have emerged with mutations in the RBD that enhance transmission and/or virulence (39–41). A critical concern in developing therapeutics targeting SARS-CoV-2 is the possibility that they will lose efficacy as the virus mutates. Indeed, the clinical efficacy of several approved mAbs against the original Washington 2020 isolate of the SARS-CoV-2 was reduced when treating patients infected with the emerging VOC (10, 42, 43). Thus, the emergence of each VOC requires reassessment of the efficacy of the therapeutics targeting the spike protein. To demonstrate that this model can be adapted to test emerging VOC, we used four different rVSV-SARS2-S viruses expressing the spike protein of B.1.1.7 (alpha, α), B.1.351 (beta, β), P.1 (gamma, γ) or B.1.617.2(delta, Δ) and compared the lethality with the corresponding wild type viruses under BSL-3 conditions. As seen in **Figure 5C**, neonatal hACE2tg mice can be lethally infected with 1 x 10^4^ TCID_50_ of rVSV-SARS2-S^B.1.1.7^, rVSV-SARS2-S^B.1.351^, rVSV-SARS2-S^P1^, or rVSV-SARS2-S^B.1.617.2^ at P5. Interestingly, both the mice infected with VOC and those infected with VSV expressing the Spike protein of the 4 VOCs showed accelerated death relative to those infected with SARS-CoV-2 (1 x 10^4^ TCID_50_) and rVSV-SARS2-S (1 x 10^5^ TCID_50_). As the only difference between these viruses is the spike protein, this underscores the critical role of the spike protein in disease severity and suggests that changes in the spike are sufficient to confer increased virulence, further studies using this model may facilitate our understanding of the role of spike protein in progression and dissemination of disease. Together, these results demonstrate that the VSV-based model can be easily adapted to emerging variants of concern and can be a useful tool to screen novel therapeutics and combinations to control new VOC as they emerge.

## Discussion

This report shows that a replicating VSV pseudovirus where the VSV-G is replaced by the Spike protein of a SARS-CoV-2 virus can be used to infect hACE2tg mice and model SARS-CoV-2 infections under BSL-2 conditions. Importantly, this virus lacks the VSV glycoprotein that mediates cell entry, so the tissue tropism is solely mediated by the engineered Spike protein, as demonstrated by the need for expression of hACE2 to infect BHK-21 cells and selective infection of hACE2 expressing mice (**Supplementary Figure 3C**). Although neonatal K18-hACE2tg mice show broad distribution of the ACE2 receptor (**Supplementary Figure 3B**), intranasal challenge with SARS-CoV-2 or rVSV-SARS2-S results in infection of the brain and, to a lesser extent, the lungs. Both challenges result in similar tissue distribution and levels of virus, with minor lung lesions and a lethal meningoencephalits. Further, both challenges increased the expression of a common subset of genes in the CNS, however the disease course is longer (8 *vs* 4 days) and the relative inflammatory gene expression levels are lower in rVSV-SARS2-S compared to SARS-CoV-2 infected mice. Lastly, testing of different mAbs targeting the spike protein resulted in similar degrees of protection in both models. Thus, this model can be used to test potential countermeasures targeting the SARS-CoV-2 spike protein in BSL-2 containment. Importantly, over the last year, several VOCs have emerged with mutations in the RBD that enhance transmission and/or virulence, and critically, can bypass the host’s memory response and reduce the effectiveness of therapeutic antibodies or vaccines that target the spike protein. Switching the spike protein on the VSV pseudotype virus allows for modeling existing or even potential mutations of the virus providing an important tool to address emerging variants.

In-line with reports showing adult C57BL/6J mice are resistant to SARS-CoV-2 infection, neither SARS-CoV-2 nor rVSV-SARS2-S were able to infect neonatal C57BL/6J mice even when deficient in type I interferons indicating that the absolute requirement for spike-hACE2 interaction for establishing infection extends to neonatal mice (14, 44). This is different from challenges with the parental VSV or from rVSV expressing the Ebola Zaire glycoproteins as previously shown (18). The increased susceptibility of neonatal mice to viral infections is well established (18, 23, 34, 35, 45, 46). This extends to coronaviruses where reports show the acquisition of virulence in young, but not aged, mice through serial passage of SARS-CoV Urbani strain (47). Consistent with this, the disease progression in neonatal mice challenged with SARS-CoV-2 was faster and lethality more consistent than that reported in adults (14, 29, 48). Surprisingly, although SARS-CoV-2 can lead to severe respiratory distress and lung pathology in adult mice, the same does not occur in neonates, where the disease affects primarily the CNS, regardless of whether they are infected with SARS-CoV-2 or rVSV-SARS2-S (29, 36). This could be partly due to the mode of infection, as intranasal infection in neonates does not entail anesthesia, to differences in the lung’s innate immune system, or the rapid lethality of the CNS infection (49). Indeed, the low level of infection in lungs compared to CNS suggests the possibility of virus uptake via the olfactory bulb rather than secondary to colonization of the lungs and hematogenous spread. In support of this, there is detectable virus in the olfactory bulb and the olfactory tract (**Supplementary Figure 6A**) as recently shown for SARS-CoV-2 and previously shown for SARS-CoV (50, 51). While no virus is evident in the olfactory bulb of rVSV-SARS2-S infected mice, this may be due to the timing of the tissue harvest relative to infection (7 dpi) as the presence of degenerate neurons suggest previous infection in the tissue. Thus, it is possible that the encephalitis, affecting particularly the brain stem and mid brain leads to lethality before high levels of virus in the lung are achieved. This would be consistent with the low level of infection and inflammation observed in the lungs and appears more likely than the low viral titers in lungs being secondary to a robust host immune response in neonates. Alternatively, it is also possible that pathogen-mediated host immune suppression contributes to the mild pathology. The lack of increase in inflammatory markers in serum late in disease course is in-line with the reduced virus RNA and inflammation seen in the lung.

Vesicular stomatitis virus (VSV), a prototype of the Rhabdoviridae family, contains a single surface glycoprotein (G) that is required for attachment to cells and mediates membrane fusion (52, 53). In the rVSV-SARS2-S model, the surface G was replaced by the SARS-CoV-2 spike protein. Several lines of evidence demonstrate that the spike-hACE2 interaction is responsible for the attachment and infection of cells: 1) *In vitro*, rVSV-SARS2-S can infect BHK21 cells stably transfected to express hACE2 receptor, but not the parental cells that do not express the receptor (**Supplementary Figure 3C**); 2) Infection of cells expressing the hACE2 receptor can be blocked by antibodies that target the spike protein (**Figure 4A**). 3) rVSV-SARS2-S cannot infect C57BL/6J or B6-IFNAR1 KO mice, but infects neonatal B6.Cg-Tg (K18-ACE2)^2Prlmn/J^ demonstrating that the interaction with hACE2 is required for infection, and 4) pre-treatment with antibodies that specifically block the spike protein protected the mice from infection (**Figure 4**). Lastly, previous studies showed that neonatal C57BL/6J mice succumb to infection when challenged with the parental VSV virus or with VSV virus where the glycoprotein (G) was replaced by the Ebola Zaire glycoprotein, however C57BL/6J mice do not succumb when challenged with VSV virus where G was replaced by the Ebola Reston glycoprotein or with SARS-CoV-2 spike protein (18). This indicates that even in susceptible neonatal mice, tissue tropism mediated by the envelope glycoprotein is required for lethal infection. Interestingly, unlike SARS-CoV-2, which can infect neonatal and adult B6.Cg-Tg (K18-ACE2)^2Prlmn/^mice, rVSV-SARS2-S infected neonatal but not adult B6.Cg-Tg (K18-ACE2)^2Prlmn/^mice. The increased susceptibility of neonatal mice is not unique (23, 35, 54). The increased susceptibility in neonates does not appear to be not due to hACE2 distribution, which is similar between neonatal and adult B6.Cg-Tg (K18-ACE2)2Prlmn/J mice (**Supplementary Figure 3B**), but could be secondary to immature innate immune responses in neonates allowing unchecked virus proliferation. Of note, previous studies have shown that neonatal mice can produce type I interferons in response to infection (18), and treatment of adult tg mice with 1600 ng of anti-IFN antibodies (SC) did not render them susceptible as no virus could be detected at 2 or 6 days post infection indicating that an immature interferon response alone is not sufficient to confer susceptibility. Other factors such as the integrity of the blood brain barrier, the frequency of immature replicating cells, or the level of other anti-viral factors such as cholesterol 25-hydroxylase (CH25H) expression (28), which has been shown to inhibit VSV and SARS-CoV-2 virus-cell fusion may contribute to the difference is susceptibility between neonates and adults (55). Given that cell tropism is determined by the spike protein, the difference in susceptibility of adult B6.Cg-Tg (K18-ACE2)^2Prlmn^ mice to challenges with SARS-CoV-2 or rVSV-SARS2-S is intriguing. These could be linked to differences in viral fitness, or potentially to small differences in binding affinity of the spike to the ACE2 receptor associated to the 21 amino acids that were excluded from the spike protein to allow for efficient virus release. Of note, despite these potential differences in the spike protein between SARS-CoV-2 and VSV-SARS2-S, the expression of spike by the VSV, allows for an induction of immune responses capable of providing protection against SARS-CoV-2 (Israely et al, 2020) suggesting presentation of the spike protein on the viral membrane with antigenic similarity to SARS-CoV-2. This is consistent with the similar levels of protection conferred by the antibody preparations with both viruses.

Although previous studies have shown that SARS-CoV-2 infection resulted in neuro invasion, this is the first report that demonstrates a SARS-CoV-2 spike protein pseudotyped, replication competent recombinant virus from a peripheral infection can spread to the CNS and result in productive infection of neurons. Understanding of the susceptibility of terminally differentiated cell types like neurons, astrocytes and microglia are difficult and often depend on *ex vivo* or *in vitro* culture of these cell types and use antigenic staining. This study uses an *in vivo* system and an enhanced GFP tag that is dependent on cellular replication to demonstrate infection of neurons and lack of replication in microglia. Neuronal infection, microglial activation and infiltration of immune cells are common to both viruses as is the increase in degenerative neurons. Unlike the lungs, the response to SARS-CoV-2 and rVSV-SARS2-S in the CNS is robust and characterized by the expected significant expression of genes linked to the type I interferon, complement activation, inflammation as well as markers of cellular infiltration (**Supplementary Figure 7**). However, the increase in regulatory markers including Il-1rn, Pdl1, Lilrb3 and Lilrb4 reflects a dynamic host immune response and suggests that the models could be used to assess combinations of anti-Spike and immunomodulatory products. Although the absolute level of individual markers is different between the models, there is striking similarity in the GO path analysis and the individual genes upregulated in the brain in both models suggesting that the rVSV-SARS2-S model can be used as effectively as the SARS-CoV-2 model to test SARS-CoV-2 countermeasures targeting the Spike protein.

Multiple studies have shown that antibodies targeting the SARS-CoV-2’s spike-ACE2 interaction are critical for protection however *in vitro* assays testing neutralization do not capture the dynamics of viral replication mediated by tissue tropism, pharmacokinetics and pharmacodynamics of therapeutics and most importantly the complexity of host-pathogen interactions. Animal models that require BSL3 containment are useful, but severely increase the cost and complexity of studies, and few facilities are set up for long-term or complex studies that can accelerate testing of therapeutic targets. While the proposed rVSV-SARS2-S model allows for easier testing, there are obvious limitations including i) the viral replication is mediated by the VSV machinery resulting in limited utility when testing tharapeutics that do not target the spike -ACE2 interaction, ii) while immunologically replete, the immune system in neonatal mice is immature, so studies assessing host-pathogen interactions or evaluating immunomodulatory agents should be interpreted cautiously, and iii) the expression of human ACE2 under the K18 promotor is likely to result in altered tissue tropism. However the model presents several advantages including specific entry and spread mediated by the SARS-CoV-2 spike protein providing a useful tool to test preventive and therapeutic strategies targeting the spike protein *in vivo* under BSL-2 conditions. This model can be used to test SARS-CoV-2 countermeasures that specifically target the spike protein, investigate the role of spike protein in mediating infection and/or antibody dependent enhancement (ADE) of disease. Indeed, the studies comparing the efficacy of different antibody preparations in blocking infection by SARS-CoV-2 and rVSV-SARS2-S illustrate the comparable utility of this VSV-based animal model. Importantly, since the host’s immune response is intact, strategies such as boosting host immune response and combination therapies that target host immune response, SARS-CoV-2 spike protein and RNA dependent RNA polymerase (RdRp) as well as the contribution of Fc mediated effector function to viral clearance can be tested in this model. Lastly, as the pandemic continues to evolve, we are facing the emergence of new variants of concern where changes to the spike protein are modifying the affinity to ACE2 and consequently the infectivity and disease severity (39). Modeling the variants using the rVSV-SARS2-S models, showed that the mutations in the spike protein led to accelerated disease progression demonstrating the critical role of SARS-CoV-2 spike glycoprotein on disease phenotype. Indeed, the plasticity of the rVSV-SARS2-S model allows not only for rapid development of animal models to address emerging variants but can also be used to model tissue tropism and test therapeutics to hypothetical variants of the spike without generating novel infectious and potentially pathogenic variants of SARS-CoV-2 in the lab.

## Data Availability Statement

The original contributions presented in the study are included in the article/**Supplementary material**. Further inquiries can be directed to the corresponding author/s.

## Ethics Statement

This study was reviewed and approved by FDA White Oak Consolidated Animal Use and Care Committee (ACUC).

## Author Contributions

DV, MoM, and DI conceived this study and drafted the Manuscript. MoM and DI performed *in vitro*, *in vivo* and *ex vivo* experiments. ST quantified serum cytokines and chemokines using Luminex multiplex assays. ME scored histology sections of mouse lungs and brain. MiM assisted with immunohistochemistry of brain tissues. SW, PR, MP and L-MB constructed rVSV-SARS2-S, rVSV-SARS2-S^α^, rVSV-SARS2-S^β^, rVSV-SARS2-S^γ^ and rVSV-SARS2^Δ^. DV provided funding, reagents and resources for the study. All authors contributed to the article and approved the submitted version.

## Funding

This study was supported in part by the Oak Ridge Institute for Science and Education through an interagency agreement between the US Department of Energy and the US Food and Drug Administration, FDA’s Medical Counter Measures Initiative (MCMi) and the Center Of Excellence in Infectious Disease and Inflammation (IDI-COE).

## Acknowledgments

The assertions herein are private ones from the authors and are not to be construed as official or reflecting the views of the US Food and Drug Administration. The authors wish to thank John Dennis, Mario Hernandez and the personnel of the Division of Veterinary Medicine for the care of the mice. We thank Andrew LaClair for BSL-3 training and guidance. We thank Carolyn Wilson, Hana Golding, and Emily Braunstein for coordinating sourcing and distribution of plasma samples, Surender Khurana for anti-SARS-CoV-2 antibodies and Tony Wang for critical SARS-CoV-2 reagents. We thank Dino Feigelstock and Christian Sauder for useful discussions and reviewing the manuscript; SARS-CoV-2 viruses including Alpha, Beta, Gamma and Delta VOC were obtained from BEI resources, NIAID, NIH. Infectious cDNA clone of SARS-CoV-2 was provided by Pei-Yong Shi (UTMB).

## Conflict of Interest

The authors declare that the research was conducted in the absence of any commercial or financial relationships that could be construed as a potential conflict of interest.

## Publisher’s Note

All claims expressed in this article are solely those of the authors and do not necessarily represent those of their affiliated organizations, or those of the publisher, the editors and the reviewers. Any product that may be evaluated in this article, or claim that may be made by its manufacturer, is not guaranteed or endorsed by the publisher.
