# Supplementary material for: BSL2-compliant lethal mouse model of SARS-CoV-2 and variants of concern to evaluate therapeutics targeting the Spike protein
Source: Front Immunol. 2022 Jul 28;13:919815. doi: 10.3389/fimmu.2022.919815 (PMC9367692; doi:10.3389/fimmu.2022.919815)
Supplement: Supplementary file 1 [file DataSheet_1.pdf]

BSL2-model paper

A replication competent VSV based BSL-2 compliant mouse model of SARS-CoV-2

Supplementary figures

Supplementary figure 1

A

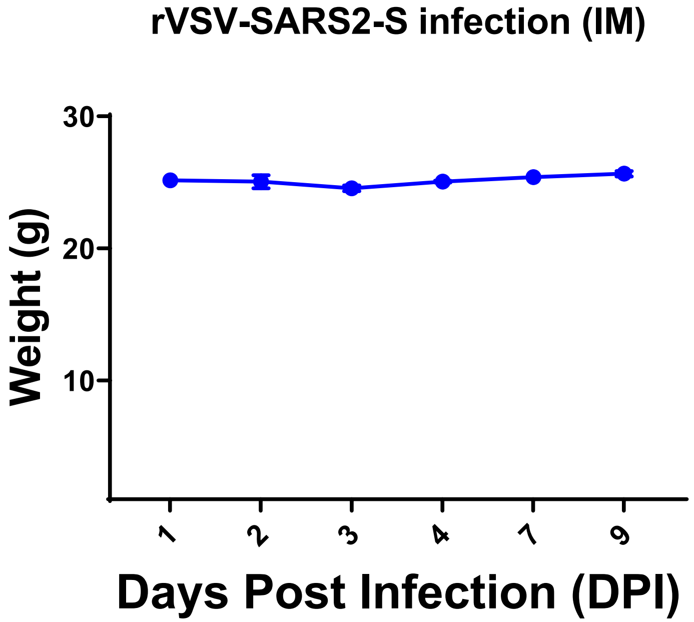

B

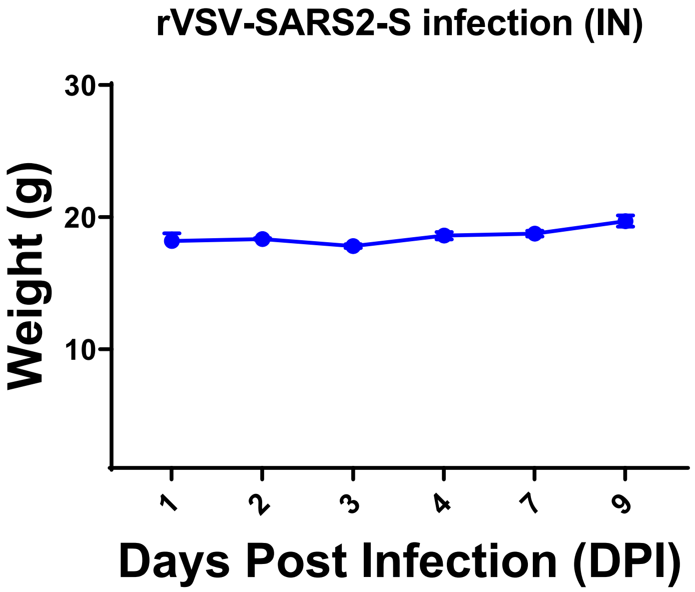

C

	**SARS-CoV2 Spike transcripts (P4)**	**TCID50 (P9)**
Blood/ serum	Negative	Negative
Lungs	Negative	Negative
Liver	Negative	Negative
Brain	Negative	Negative

S.1. **Adult C57BL/6J mice are not susceptible to rVSV-SARS2-S infection. A&B.** Intramuscular (A) or Intranasal (B) infection with 10^5 $TCID_{50}$ rVSV-SARS2-S did not result in weight loss or observable symptoms in C57BL/6J mice. **C.** Table showing the absence of viral spike protein transcripts measured by SARS-CoV-2 spike protein specific Taqman assay (at P4) and absence of infectious virus measured by Tissue Culture Infectious Dose 50 ($TCID_{50}$) assay in Vero E6 cells (at P9) from blood, lungs, liver and brain homogenates of infected C57BL/6J mice. For each condition tested, n= 3-5 mice;

Supplementary figure 2

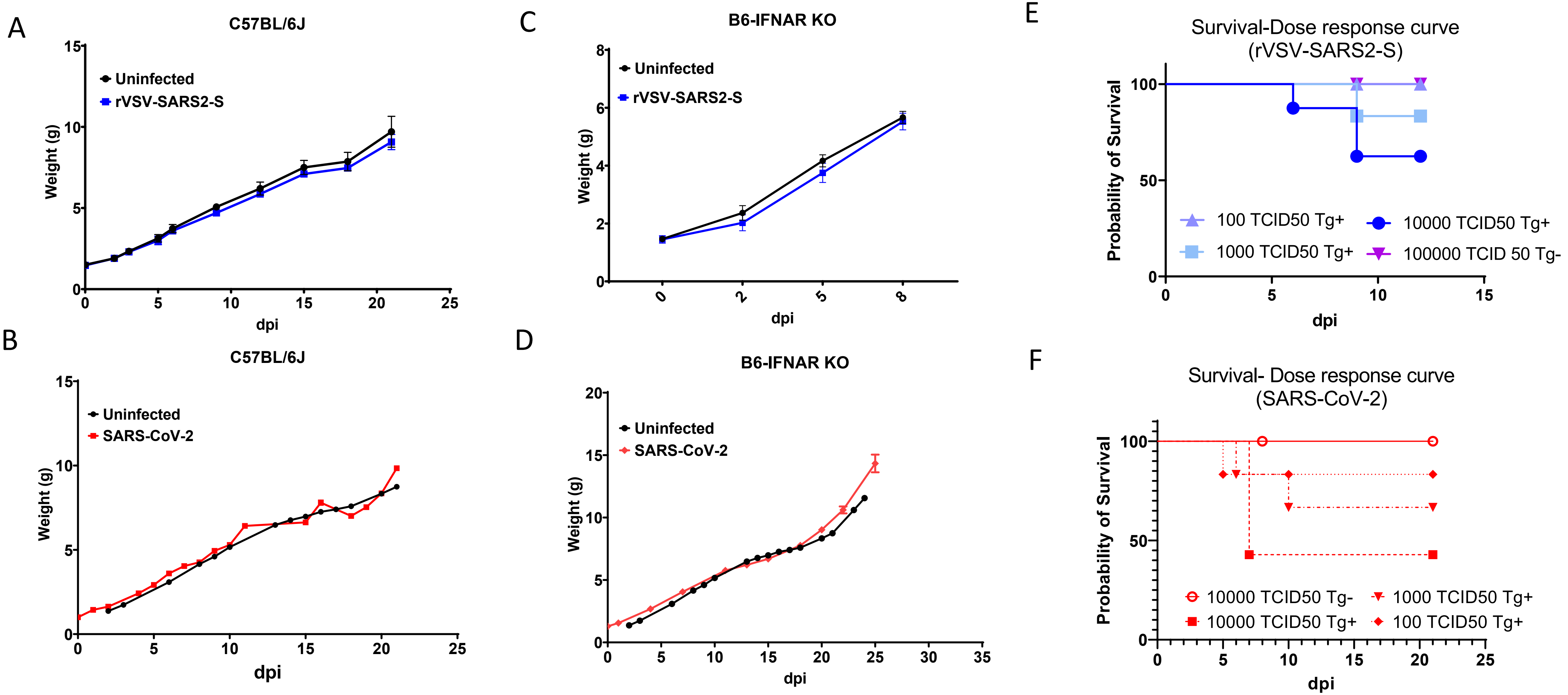

S.2. **Neonatal C57BL/6J and B6 IFNAR KO mice are resistant to SARS-CoV-2 and rVSV-SARS2-S infection. A,B,D&E**. Neonatal C57BL/6J mice (A&B) and B6-IFNAR KO mice (C&D) were intranasally infected with 10^5 $TCID_{50}$ of rVSV-SARS2-S on P1 (A&C) or 10^5 $TCID_{50}$ of SARS-CoV-2 on P5 (B&D) and monitored for weight gain. **E&F.** Human ACE2 transgenic mice were intranasally infected with 10^2, 10^3 or 10^4 $TCID_{50}$ of rVSV-SARS2-S (E) or SARS-CoV-2 (F) and monitored for survival. Tg+ refers to human ACE2 transgenic mice and Tg- refers to C57BL/6J mice. At least 5 mice were used for each condition tested.

Supplementary figure 3

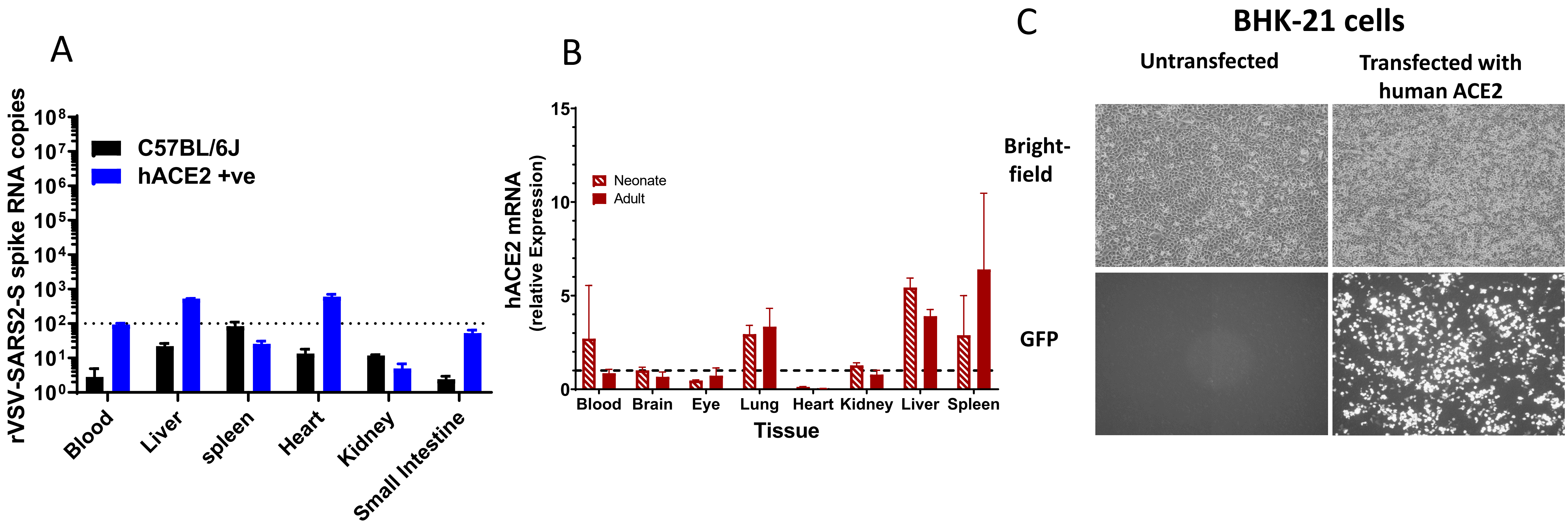

S.3. **Expression of human ACE2 is essential but may not be sufficient for SARS-CoV-2 spike protein mediated infection.** A. Human ACE2 transgenic (Blue bars) and C57BL/6J (black bars) were infected with 10^5 $TCID_{50}$ of rVSV-SARS2-S IN. At 8 dpi, viral titers were measured using SARS-CoV-2 spike protein specific Taqman assay in the blood, liver, spleen, kidney, and small intestine homogenates of rVSV-SARS2-S infected (n=3-5/group) hACE2tg mice. Horizontal line indicates the limit of detection. B. Expression of human ACE2 mRNA in tissues from neonatal (P1) and adult (9 weeks old) K18-hACE2tg mice. The levels of mRNA are relative to the mean levels of hACE2 in the brain of neonatal mice (n=3 / group). C. BHK-21 cells, untransfected (left) or stably transfected with human ACE2 (right) were challenged with rVSV-SARS2-S at a 0. 1 MOI. Brightfield (top) and fluorescence (bottom) at 24h show that the virus requires hACE2 expression to infect the cells.

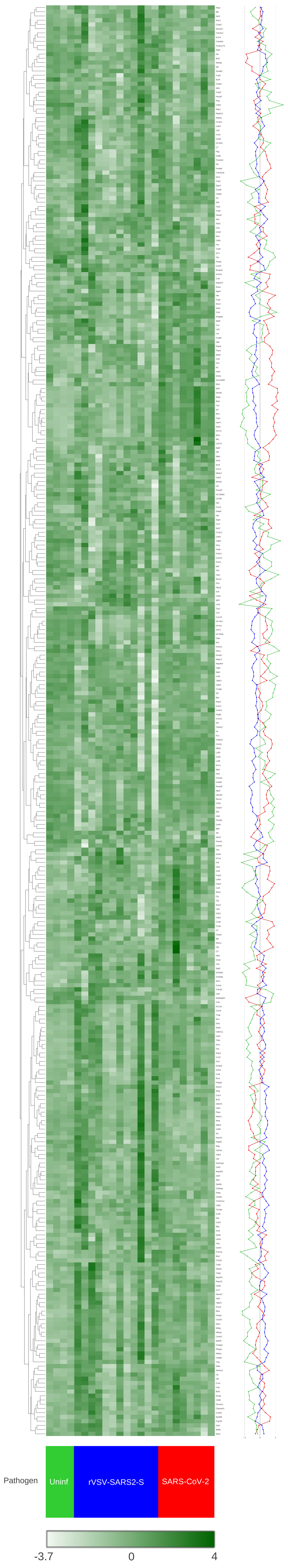
Pathogen
Uninf
rVSV-SARS2-S
SARS-CoV-2
-3.7
0
4

Supplementary figure 4 Complete gene expression data set Lungs

S.4. **Gene expression in the lung**: Complete profile of 547 immunology-related mouse genes (Nanostring- nCounter mouse immunology panel) used for gene expression analysis of infected lung tissue presented in Fig 2B. Gene expression was normalized on positive controls and housekeeping genes. Signal is the standardized values of all sample’s log-scaled counts across a given gene.

Supplementary figure 5

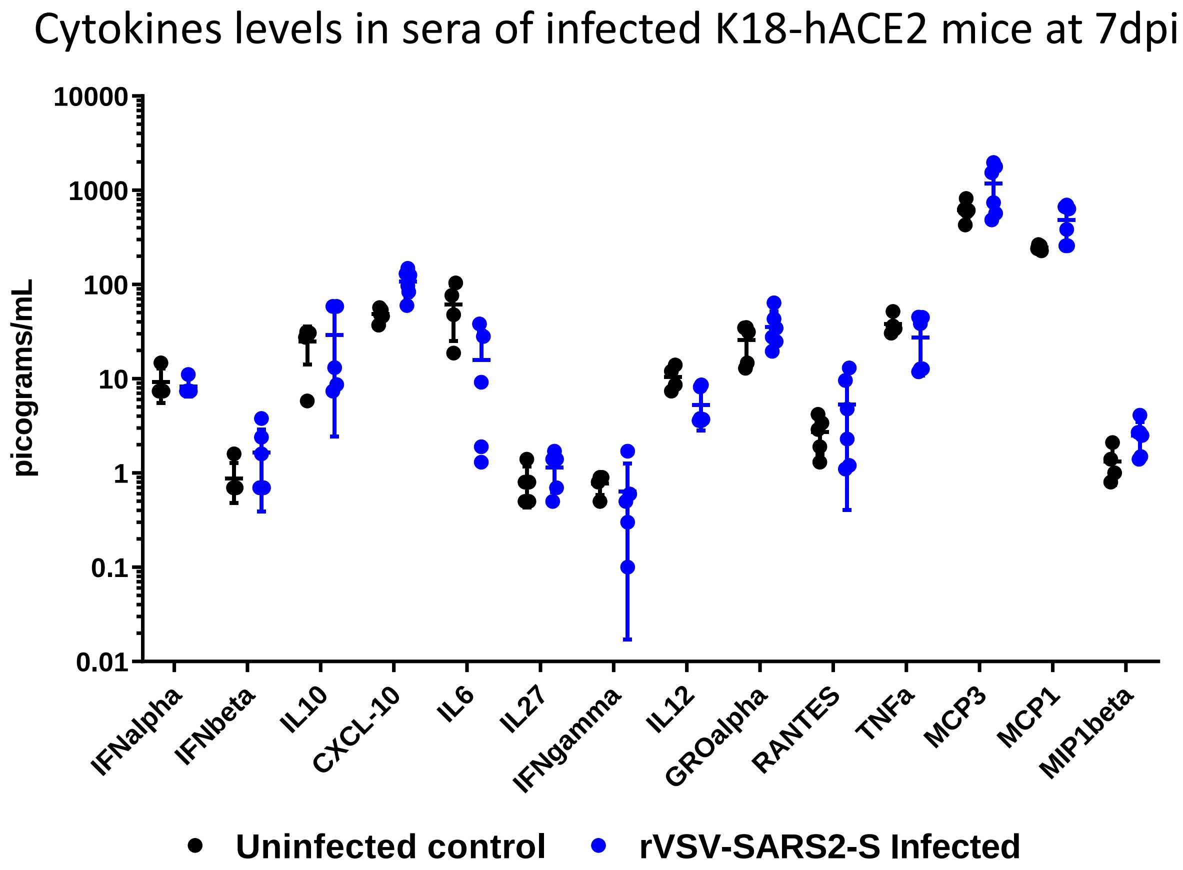

S.5. **Cytokine and Chemokine secretion in the serum of rVSV-SARS2-S infected hACE2tg mice.** Levels of cytokines and chemokines in the serum of K-18 hACE2tg mice infected (p1) with 10^5 $TCID_{50}$ of rVSV-SARS2-S (Blue) and uninfected (Black) at 7 dpi were assessed using Luminex multiplex assay as per manufacturers instructions (Thermo ProcartaPlex kits; ThermoFisher, Carlsbad, CA).

Supplementary 6A

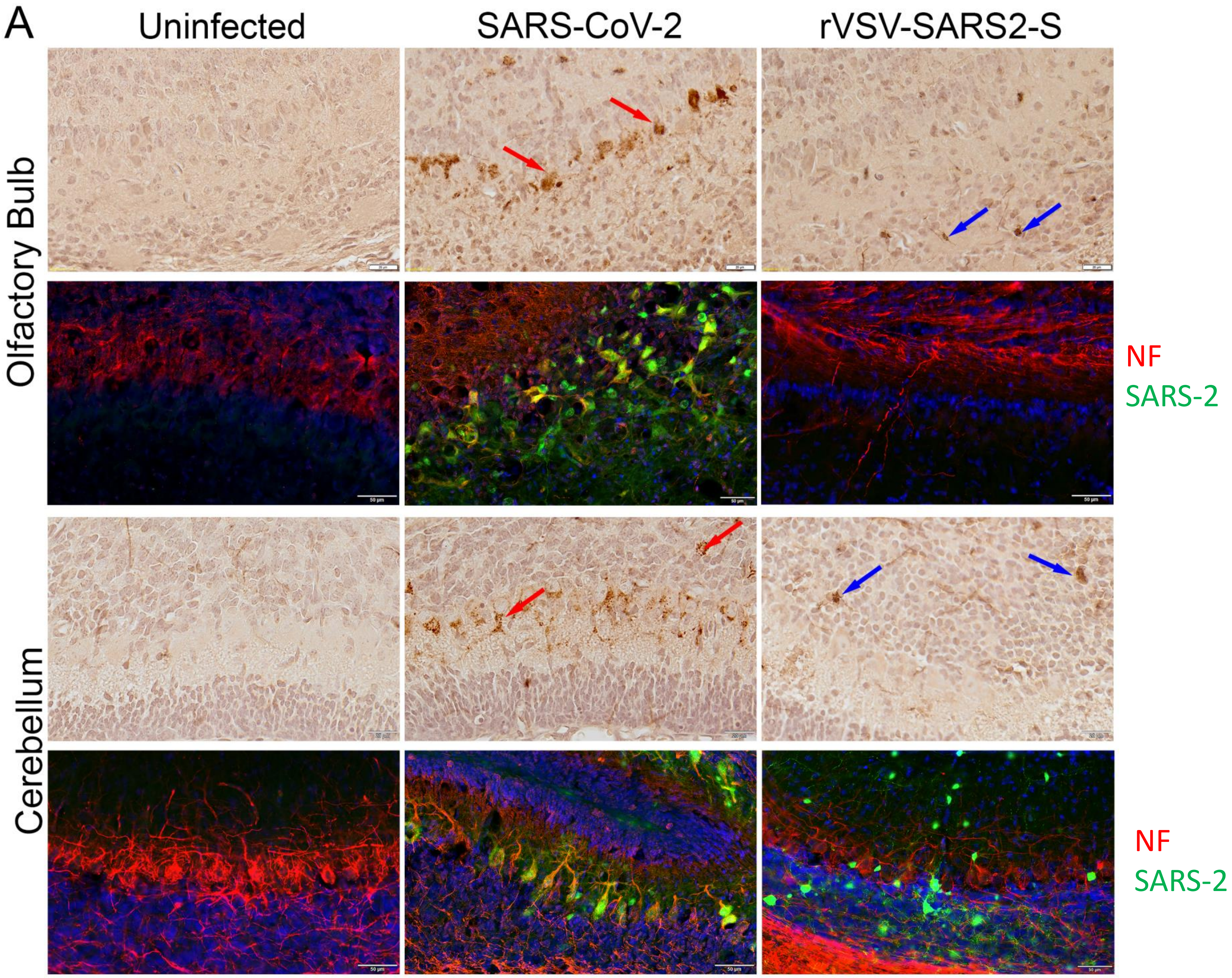

Supplementary 6B – neuronal infection

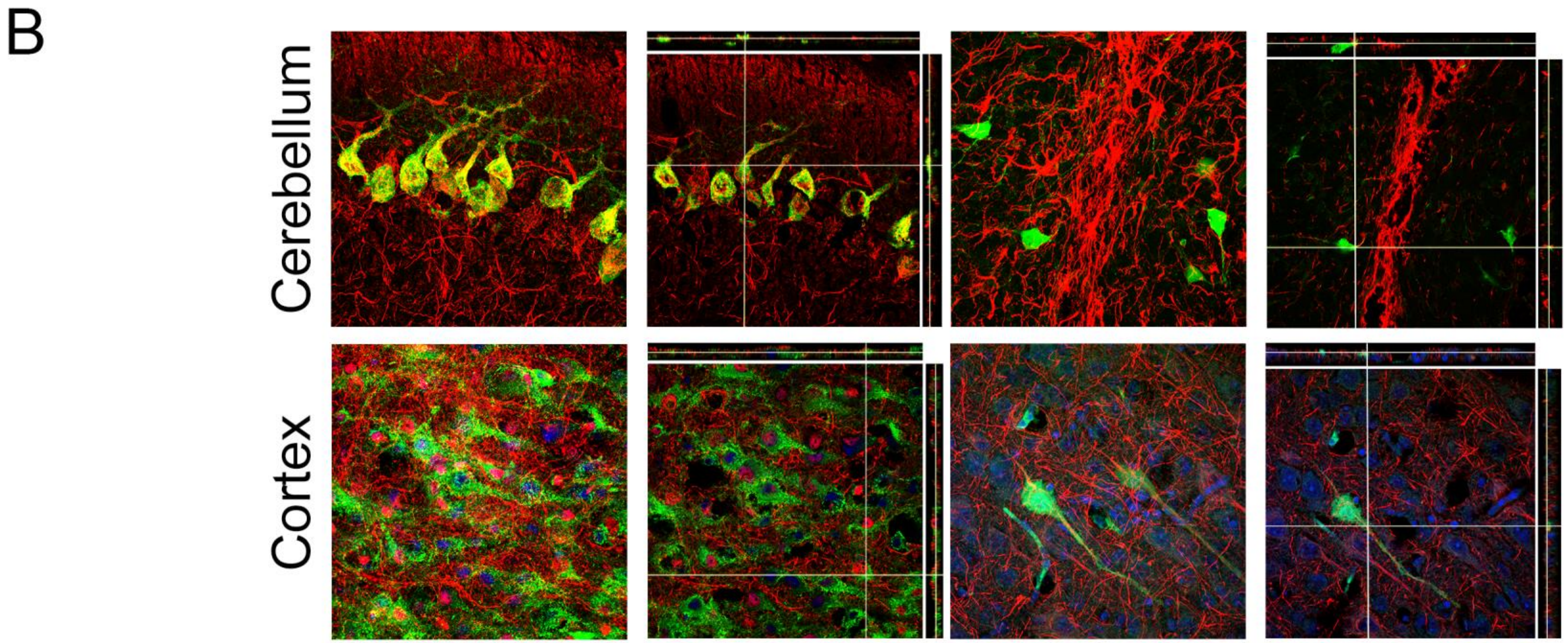

S.6. **A.** Infection of neurons in the Olfactory Bulb (OB) and Cerebellum. Top: IHC for Spike RBD indicated infection of the mitral cell layer by SARS-CoV-2 (red arrows) where only modest infection was observed in the glomerular layer with rVSV-SARS2-S infection. Similarly, GFP expressed by SARS-CoV-2 (green) was observed in the mitral and glomerular layers, whereas GFP was not observed in rVSV-SARS2-S infected OB. Colocalization with NF (red) confirms infected neurons in the OB follow SARS-CoV-2 infection. Bottom: SARS-CoV-2 (red arrows) infection was primarily observed in the Purkinje cell layer by IHC and GFP expression and co-localization with NF (red). Interestingly, rVSV-SAR2-S infected neurons were primarily found in the granular molecular layers only, with rare or absent infection of Purkinje cells. **B.** 3D Confocal microscopy confirms neuronal infection. Maximum intensity projections of Z-stack confocal images. GFP expression (green) marks infected cells, NF (red) indicates neurons and neurites. Colocalization is confirmed by orthogonal views of the 3D stacks.

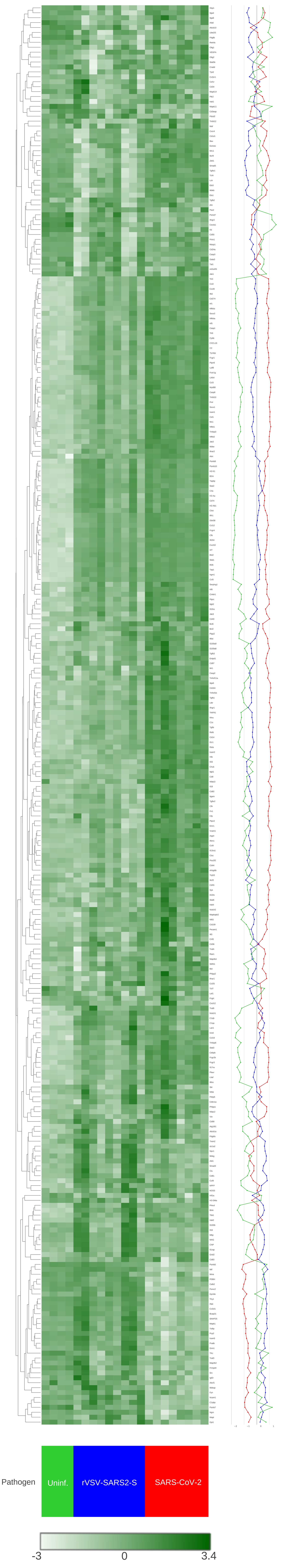
Pathogen
Uninf.
rVSV-SARS2-S
SARS-CoV-2
-3
0
3.4

Supplementary figure 7 Complete gene expression data set: Brain

S.7. **Gene expression in the brain**: Complete profile of 547 immunology-related mouse genes (nanostring-nCounter mouse immunology panel) used for gene expression analysis of infected brain tissue presented in Fig 3C. Signal is the standardized values of all sample’s log-scaled counts across a given gene.

Supplementary figure 8

Comparison of pathways regulated by SARS-CoV2 vs rVSV-SARS2-S virus infection in the brain

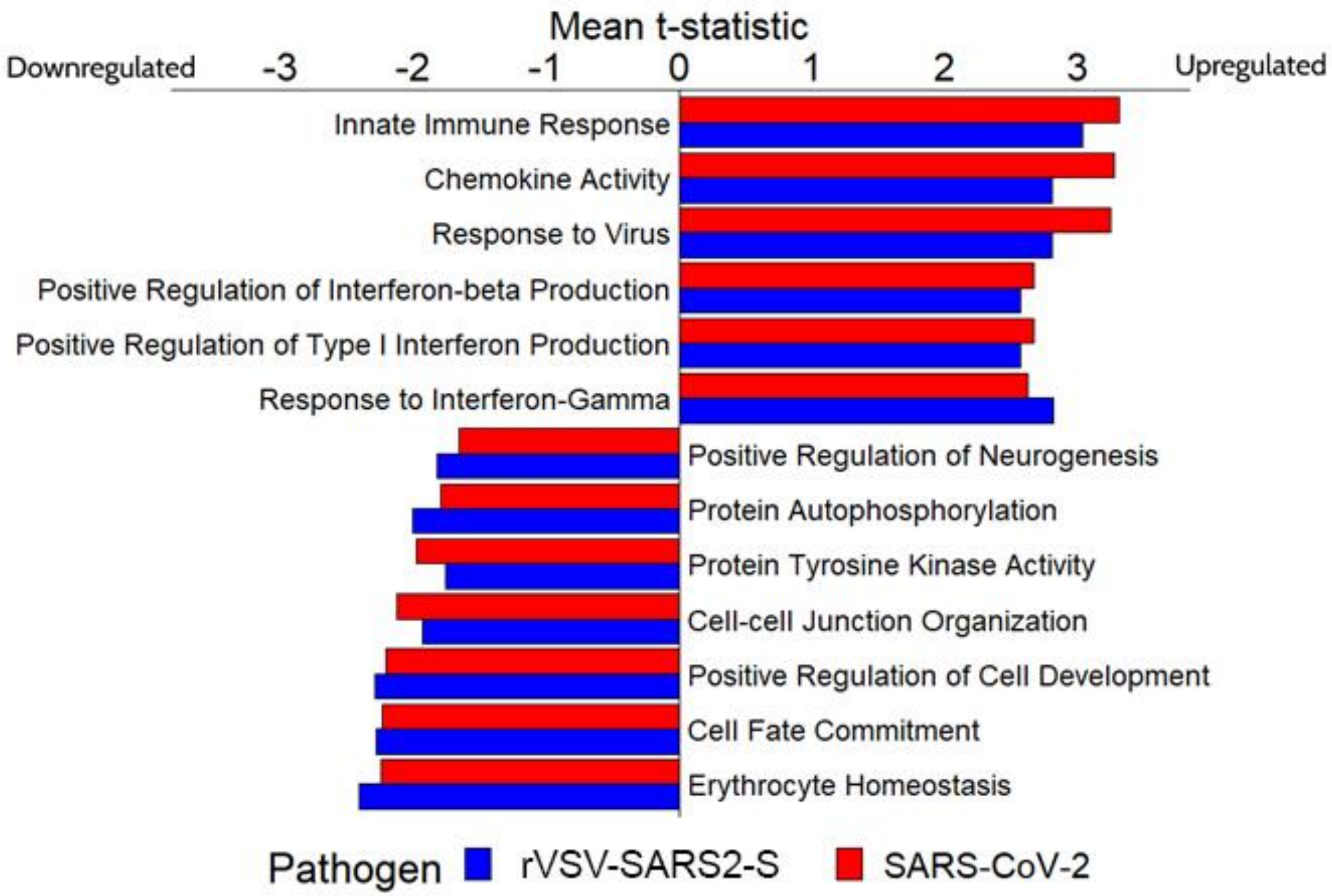

S.8. **Pathway analysis:** Gene expression profiles regulated by the infection of SARS-CoV-2 (red) and rVSV-SARS2-S(blue) in the CNS were categorized into different pathways and a comparison of upregulated and downregulated pathways in response to the infection are presented.

Supplementary figure 9

Effect of therapeutic CV30 mAb on survival of K18-hACE2Tg mice

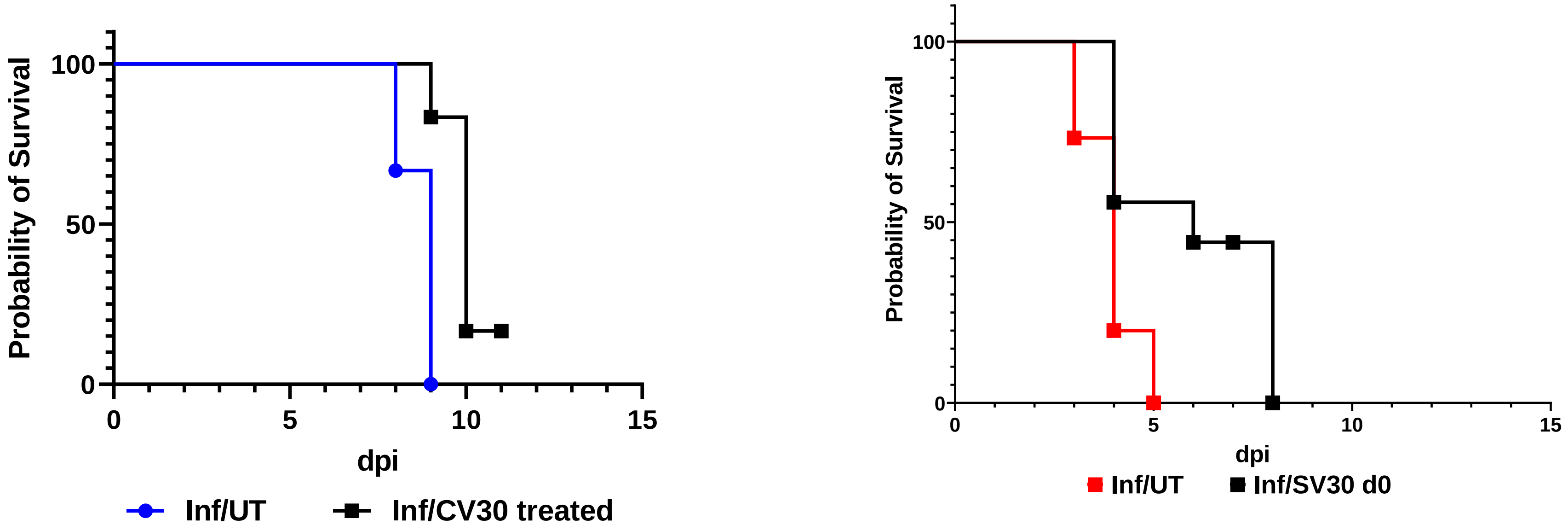

S.9. Effect of mAb treatment 24 h post infection. Human ACE2tg mice were treated intraperitoneally with 2µg/g of CV30 mAb (black line) 24 hours after intranasal infection with 10^5 $TCID_{50}$ of rVSV-SARS2-S (n=6) or SARS-CoV-2 (n=7) and monitored for survival. Untreated, age matched SARS-CoV-2 (red line, n=7) and rVSV-SARS2-S (blue line, n=3) Infected hACE2tg mice were used as controls.
